# Effects of highly active antiretroviral therapy and its adherence on herpes zoster incidence: a longitudinal cohort study

**DOI:** 10.1186/1742-6405-10-34

**Published:** 2013-12-27

**Authors:** Chenglong Liu, Cuiwei Wang, Marshall J Glesby, Gypsyamber D’souza, Audrey French, Howard Minkoff, Toby Maurer, Roksana Karim, Mary Young

**Affiliations:** 1 Department of Medicine, Georgetown University, 2115 Wisconsin Avenue NW, Suite 130, Washington, DC 20007, USA; 2 Division of Infectious Diseases, Weill Cornell Medical College, New York, NY, USA; 3 Department of Epidemiology, Johns Hopkins School of Public Health, Baltimore, MD, USA; 4 Division of Infectious Diseases, Cook County Hospital, Chicago, lL, USA; 5 Department of Obstetrics and Gynecology, Maimonides Medical Center, Brooklyn, NY, USA; 6 Department of Dermatology, University of California at San Francisco, San Francisco, CA, USA; 7 Departments of Pediatrics and Preventive Medicine, University of Southern California, Los Angeles, CA, USA

**Keywords:** HAART, Adherence, Herpes zoster, Incidence, Propensity score

## Abstract

**Background:**

Herpes zoster (HZ) is common among HIV-infected individuals, but the impacts of highly active antiretroviral therapy (HAART) and HAART adherence on HZ risk have not been well studied.

**Methods:**

The effects of HAART and HAART adherence on HZ incidence were evaluated by comparing HIV-infected women on HAART (HAART use group) with the HIV-infected women remaining HAART naïve (HAART naïve group) in the Women’s Interagency HIV Study (WIHS). A 1:1 matching with propensity score for predicting HAART initiation was conducted to balance background covariates at index visit, including HIV disease stage. Kaplan-Meier method was used to compare the risk of HZ development between the matched pairs. Cox proportional hazard models were used to assess the effects of HAART and HAART adherence on HZ incidence.

**Results:**

Through propensity score matching, 389 pairs of participants were identified and they contributed 3,909 person years after matching. The background covariates were similar between the matched pairs at the index visit. The participants had a mean age around 39 years old, and about 61% of them were Black and 22% were Latina. No significant difference in HZ risk was observed between the HAART use group and the HAART naïve group during the first year of follow-up in any analyses. In the univariate analysis, the HAART use group had marginally lower HZ risk (Hazard Ratio (HR): 0.72; 95% Confidence Interval (CI): 0.48-1.1) over the entire follow-up period. However, women with a HAART adherence level of ≥95% had significantly lower HZ risk (HR: 0.54; 95% CI: 0.31, 0.94) compared to the HAART naïve women. The association remained significant after adjusting for quality of life score and acyclovir use, but it attenuated and was no longer statistically significant after adjusting for an intermediate variable, either CD4+ T cell counts or HIV viral load.

**Conclusions:**

Among adult women, we observed a significant preventive effect of long-term HAART use on HZ incidence when a HAART adherence level of ≥95% was attained, and this effect was mediated through reduction of HIV viral load and improvement of CD4+ T cell counts.

## Background

Highly active antiretroviral therapy (HAART) has been demonstrated to be very effective in decreasing the incidence of opportunistic infections, acquired immunodeficiency syndrome (AIDS) and death among HIV-infected patients. However, previous studies showed that the incidence of herpes zoster (HZ) is less likely than those of other opportunistic infections to be reduced by HAART as it still occurs at a relatively high rate in the HAART era [1-3].

According to studies conducted so far, the effect of HAART on HZ incidence may differ depending on how long HAART has been used. Martinez et al. first reported a high HZ incidence in patients with AIDS soon after initiation of therapy with protease inhibitors [4]. Later, many other studies confirmed their observation as part of an immune reconstitution inflammatory syndrome [5-15]. It was proposed that this transient increase of HZ risk may be associated with immunodysregulation rather than profound immunesuppression. However, other studies showed no association of HZ incidence with recent HAART initiation [2,16].

The long-term effect of HAART on HZ incidence has also been inconsistent across studies. One study reported that the HZ incidence did not change between the pre-HAART era and the HAART era, and that being on HAART was associated with a high risk of HZ [16]. Furthermore, there are anecdotal reports of failure of antiretroviral therapy to control HZ complications, such as varicella zoster retinitis [17]. However, many other studies showed a protective effect of HAART on the incidence of HZ [18-21] and its complications [16,22]. Unfortunately, most of these studies did not take into account HAART adherence, which might partially explain the inconsistent results observed.

The objective of our study was to examine the effects of HAART and HAART adherence on HZ incidence by comparing HIV-infected women on HAART as a group (HAART use group) or stratified by HAART adherence level, with the matched HIV-infected HAART naive women (HAART naïve group) using longitudinal data from the Women’s Interagency HIV Study (WIHS).

## Methods

### Study population

The WIHS was initiated in 1993/1994 and expanded in 2001/2002 to examine the natural and treated history of HIV disease among women. The study design of the WIHS is detailed elsewhere [23,24]. Briefly, 2,813 HIV-infected and 953 high risk HIV-negative women were recruited from six study sites (Chicago, Los Angeles, San Francisco, Washington D.C., Brooklyn and the Bronx) in the United States. Women visit a study site every 6 months. During each visit, structured interviews are performed to collect data on socio-demographical characteristics, sexual behaviors, substance use, health care utilization, antiretroviral therapy, treatments of comorbidities and different disease outcomes. In addition, physical and obstetric/gynecologic examinations are performed and biological specimens are collected. The local institutional review board at each site has approved the study protocol, and all women have given their written informed consents. For this study, our analyses were restricted to the HIV-infected participants only.

### Propensity score matching

Unlike in randomized clinical trials, use of therapy in observational studies is not based upon random assignment. Thus, unbalanced distributions of background covariates may bias estimated treatment effects. To account for this, a propensity score matching method was used in our analysis. This method approaches more closely a randomized clinical trial by balancing background covariates via matching propensity score, which is the probability of HAART initiation in our study. The propensity score was calculated for each subject at each of her visits before starting HAART using a logistic regression model, with potential confounders for HAART initiation as predictors, including age, race, education, AIDS status, CD4+ and CD8+ T cell counts, HIV viral load and quality of life score. Each HAART user was matched at the last visit before her HAART initiation to a visit of a HAART naïve participant with similar propensity score (±0.1%). For any HAART naïve participant already selected as a control, the rest of her visits were removed from the matching pool to ensure 1:1 matching ratio. Participants who had HZ history on or before the matched visits were also removed from the analysis. The matched visits are the index visits after which all follow-up occurs in this analysis.

### Study variables

We used a longitudinal cohort design to assess the impact of HAART use and its adherence on HZ incidence. In the WIHS, questions about HZ occurrence were asked from visit 1 (October 1994) to visit 30 (April 2009). At WIHS study enrollment, participants were asked “Has a health care provider ever told you that you had shingles (herpes zoster)?” At follow up visits, participants were asked “Since your last visit, has a health care provider told you that you had shingles (herpes zoster)?” The study outcome was time to first HZ event from the index visit and participants were censored at the last study visit, death, or loss to follow up. As a participant usually had difficulty in recalling exact date of a HZ event, we used a visit date as the HZ event date when HZ was first reported at that visit, in our calculation of time to first HZ event. The exposure variables were HAART use group (yes/no) and HAART adherence level. The definition of HAART in the WIHS was guided by the DHHS/Kaiser Panel guidelines [25]. The HAART use group was defined as the matched HAART using women, while the HAART naïve group was defined as the matched women who never initiated HAART during the follow-up. HAART adherence level was self-reported and at each visit participants were asked the extent to which they took antiretrovirals as prescribed over the past 6 months. Participants categorized their level of adherence into one of five categories: 100% of the time, 95-99% of the time, 75-94% of the time, <75% of the time, and have not taken any prescribed medications. In WIHS, a shortened version of MOS-HIV developed by Bozzette et al. was adopted to measure QOL and its summary score was used [26]. The score ranged from 0–100, with higher score indicating better QOL. Time varying covariates were selected based on causal diagram analysis of factors that might affect HZ incidence; CD4+ T cell counts, CD8+ T cell counts and HIV viral load were potential intermediate variables, while quality of life score and acyclovir use since the last study visit were potential confounders.

### Statistical analysis

Descriptive statistics such as mean and proportions were used to assess distributions of background covariates before and after propensity score matching. Kaplan-Meier method was used to describe HZ free curves during study follow up. Cox proportional hazards models were used to estimate associations of risk factors with time to first HZ event from the index visit, as reflected by hazard ratios (HR) and their corresponding 95% confidence intervals (CI). The underlying proportional hazard assumption in the Cox models was assessed for each covariate, and we found the assumption was satisfied, with the smallest P value being 0.2959. Values of time varying variables at the last study visit were excluded from analyses to avoid temporal ambiguity. For example, acyclovir use that was reported at the visit at which HZ was first reported was not counted as acyclovir use prior to HZ since the drug may have been prescribed to treat HZ. Univariate associations were first evaluated and only variables significant in univariate analysis were included in multivariate models. For short-term HAART effect, the study was assumed to end one year after the index visit. For long-term HAART effect, the participants were followed from the index visits to either their first HZ event or censored events such as death, loss to follow up or at the last visit (visit 30). To assess the effect of HAART use on HZ incidence, we assumed that a woman who initiated HAART will continue on HAART, which is similar to an intent-to-treatment analysis in a clinical trial. To assess the effect of HAART adherence on HZ incidence, time varying HAART adherence level was used as exposure variable, which is similar to the as-treated analysis in a clinical trial. To examine whether HAART exerts its effect via immune recovery, we purposely added into the multivariate models an intermediate variable between HAART use and HZ incidence, along with an exposure variable and potential confounders. All statistical analyses were carried out using SAS 9.2 (SAS Inc, Carey, NC).

## Results

### Trends in HAART use and HZ incidence in the WIHS

**Figure 1:**
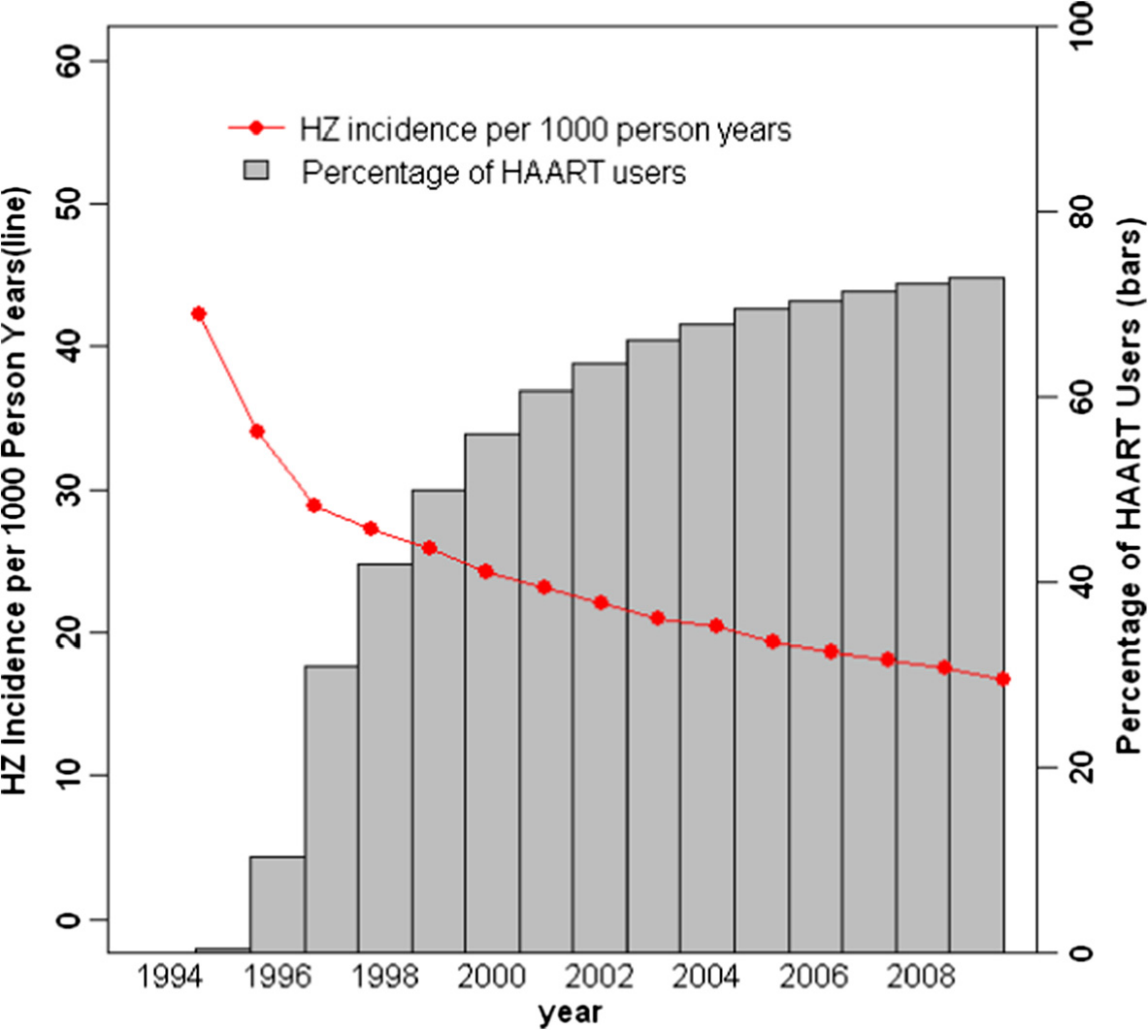
Trends in percentage of HAART users and herpes zoster Incidence among HIV-infected women in the WIHS.

2,813 HIV-infected WIHS women were followed from the WIHS enrollment until visit 30 (2009) and contributed 22,658 person years. Figure 1 shows trends in HAART use and HZ incidence among these women during this period. HZ incidence decreased substantially during the first few years and it declined steadily thereafter, coincident with an increase in the proportion of HIV-infected women taking HAART.

### Propensity score matching and participant characteristics balancing

Characteristics of all HAART using women (2053) and all HAART naïve women (760) at the WIHS enrollment, and characteristics of the subsets of these women who were matched by propensity score at the index visit are presented in Table 1. At the WIHS enrollment, distributions of age, race, AIDS diagnosis, HIV viral load, CD4+ and CD8+ cell counts were significantly different between HAART use and HAART naïve women. However, after propensity score matching, all background covariates of the 389 matched pairs were well balanced at the index visit.

**Table 1:** Characteristics comparison before and after matching

**Covariates**	**Before matching at WIHS**^**b **^**enrollment**			**After matching at index visit**		
	**All HAART**^**c **^**use women**	**All HAART naïve women**	**p-Value**	**Matched HAART use women**	**Matched HAART naïve women**	**p-Value**
	**(N = 2053)**	**(N = 760)**		**(N = 389)**	**(N = 389)**	
**Age, mean (SD**^**a**^**)**	35 (7.9)	37 (7.6)	*<.0001*	39.7 (8.7)	39.4 (8.2)	0.57
**Race, N (%)**			*0.015*			0.81
**White, non-hispanic**	308 (15.0)	122 (16.1)		57 (14.7)	52 (13.4)	
**Black, non-hispanic**	1119 (54.5)	451 (59.3)		233 (59.9)	246 (63.2)	
**Hispanic**	560 (27.3)	162 (21.3)		88 (22.6)	80 (20.6)	
**Other**	66 (3.2)	25 (3.3)		11 (2.8)	11 (2.8)	
**Education, N (%)**			0.086			0.64
**Less than high school**	759 (37.1)	304 (40.1)		148 (38.1)	158 (40.6)	
**Completed high school**	612 (29.9)	237 (31.2)		126 (32.4)	127 (32.7)	
**Some college and above**	677 (33.1)	218 (28.7)		115 (29.6)	104 (26.7)	
**AIDS, N (%)**	407 (19.8)	308 (43.08)	*<.0001*	135 (34.7)	138 (35.5)	0.82
**CD4+ cell counts, mean (SD)**	430 (291.7)	398 (365.3)	*<.0001*	372.6 (248.1)	358.7 (279.2)	0.47
**CD8+ cell counts, mean (SD)**	899.7 (464.1)	792.2 (597.3)	*<.0001*	847.2 (519.3)	806.8 (521.4)	0.28
**log10 (Viral load), mean (SD)**	3.9 (1.1)	4.4 (1.2)	*<.0001*	3.9 (1.1)	4.1 (1.2)	0.14
**Quality of life score, mean (SD)**	64.8 (20.4)	57.4 (21.2)	0.18	63.8 (21.5)	62.4 (22.2)	0.35

^a^standard deviation; ^b^WIHS: Women’s Interagency HIV Study; ^c^HAART: highly active anti-retroviral therapy.

### Comparison of HZ free curves between the HAART use group and HAART naïve group

**Figure 2:**
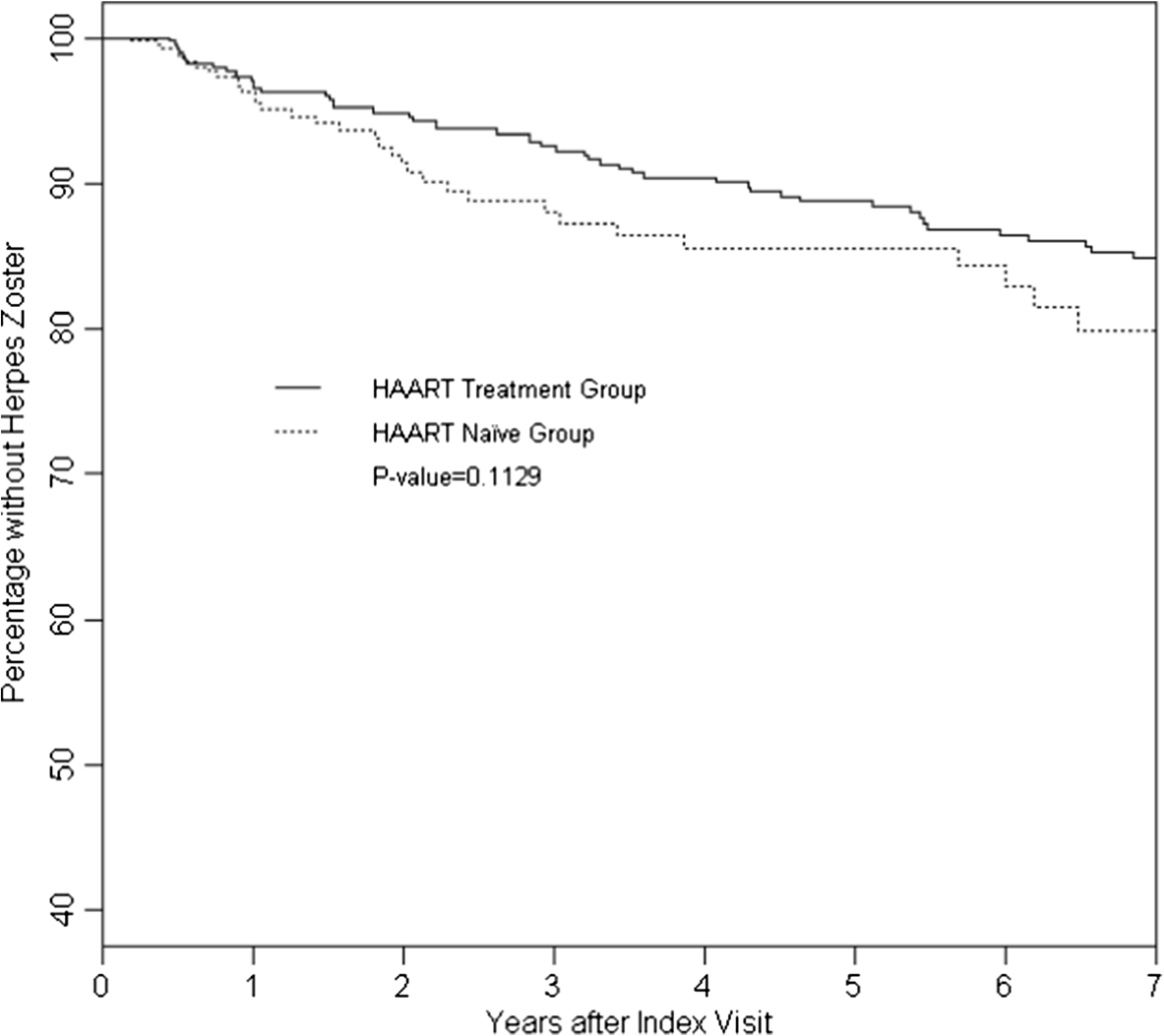
Percentage without herpes zoster for HAART treatment and HAART naïve group.

During the follow up, the 389 matched pairs contributed 3,909 person years and reported 109 HZ events. The HAART use group had a median follow-up time of 6.9 years (interquartile range (IQR: 25th percentile to 75th percentile): 3.5-11 years) and an average HZ incidence of 25 (95% confidence interval(CI): 20–32) per 1000 person years. The HAART naïve group had a median follow-up time of 1.5 years (IQR: 0.7-3.8 years) and an average HZ incidence of 35 (95% CI: 26–48) per 1000 person years Figure 2 describes the cumulative probability of remaining HZ free (i.e. not developing HZ) for the HAART use group and the HAART naïve group. During the first year of follow up, HZ-free survival was similar. However, with additional follow-up, the difference started showing up. Overall, the HAART naïve group had marginally lower HZ-free survival (i.e. a higher incidence of HZ) over time than the HAART use group (*P* = 0.113).

### Short-term Effect of HAART on HZ incidence

During the first year of follow up, the HAART use group and the HAART naïve group reported 13 (incidence rate: 49 per 1000 person years; 95% CI (29, 82)) and 12 (incidence rate: 47 per 1000 person years, 95% CI (27, 81)) HZ events respectively. In the univariate analysis, compared to the HAART naïve group, neither the HAART use group (HR: 0.88; 95% CI: 0.39-1.99) nor women with a HAART adherence level of ≥95% (HR: 1.20; 95% CI: 0.42-3.11) had significantly different risk of HZ incidence.

### Long-term Effects of HAART on HZ incidence

Effects of long-term HAART use and HAART adherence on HZ incidence are described in Table 2. In univariate analysis, the HAART use group had a marginally lower HZ incidence (HR: 0.72; 95% CI: 0.48-1.09) compared to the matched HAART naïve group. However, women with a HAART adherence level of ≥95% had significantly lower HZ incidence (HR: 0.54; 95% CI: 0.31-0.94) than HAART naïve individuals, while women with lower HAART adherence levels (less than 95%) or HAART discontinuation had similar HZ risk to that of the HAART naïve group. Higher CD4+ T cell counts (per 100 increase; HR: 0.85; 95% CI: 0.77-0.93) was significantly associated with lower HZ incidence while higher HIV viral load (per log_10_ increase; HR: 1.55; 95% CI: 1.30-1.84) was significantly related to higher HZ incidence. After adjusting for QOL score and acyclovir use, a HAART adherence level of ≥95% remained significantly associated with lower HZ incidence. However, after adding an intermediate variable - either CD4+ T cell count or HIV viral load, along with QOL score and acyclovir use, the association of a HAART adherence level of ≥95% with HZ incidence attenuated and was no longer statistically significant.

**Table 2:** **Long-term effect of haart**^**a **^**use on herpes zoster incidence**

**Variable**	**Univariate**	**Multivariate**^**1**^	**Multivariate**^**2**^	**Multivariate**^**3**^
	**HR**^**b **^**(CI**^**c**^**)**	**HR (CI)**	**HR (CI)**	**HR (CI)**
**HAART use group:**				
**No**	1.00			
**Yes**	0.72(0.48–1.09)			
**HAART use adherence since last visit**^**c**^				
>95%	0.54*(0.31–0.94)	0.56*(0.32–0.99)	0.61(0.33–1.11)	0.76(0.41–1.38)
75%–95%	0.32(0.09–1.05)	0.31(0.09–1.03)	0.30(0.09–1.03)	0.34(0.10–1.14)
0 < adherence <75%	1.70(0.70–4.14)	1.38(0.53–3.63)	1.25(0.46–3.41)	1.23(0.46–3.31)
HAART users, but did not use HAART since last visit	0.95(0.52–1.72)	0.87(0.47–1.62)	0.77(0.39–1.52)	0.65(0.33–1.27)
No HAART users	1.00	1.00	1.00	1.00
**Current CD4+ cell counts (per100 cell increase)**^**d**^	0.85***(0.77–0.93)		0.86***(0.78–0.94)	
**Current CD8+ cell counts (per 100 cell increase)**^**d**^	0.96(0.90–1.01)			
**HIV viral load (per Log 10)**^**d**^	1.55***(1.30–1.84)			1.59***(1.31–1.93)
**Quality of life score**^**d**^	0.98**(0.97–0.99)	0.99*(0.98–0.99)	0.98*(0.97–0.99)	0.98*(0.97–0.99)
**Used acyclovir since last visit**^**d**^**:**				
**No**	1.00	1.00	1.00	1.00
**Yes**	1.88*(1.04–3.18)	1.68(0.88–3.22)	1.52(0.75–3.10)	1.56(0.79–3.08)

^a^HAART: highly active antiretroviral therapy; ^b^hazard ratio; ^c^95% confident interval; ^d^time dependent variables ***p < 0.001; **0.001 ≤ p < 0.01; *0.01 ≤ p < 0.05; ^1^after adjusting for quality of life and acyclovir use; ^2^after adjusting for quality of life, acyclovir use and CD4+ T cell counts; ^3^after adjusting for quality of life, acyclovir use and HIV viral load.

## Discussion

Though the mechanisms by which HAART exerts its effect on HZ incidence are not yet clear, accumulating evidence suggests that HAART might have different effects on HZ incidence depending on how long HAART has been used. Many studies demonstrated that HZ occurs at a high level shortly after initiation of HAART [4-15], which might reflect dysregulated immune responses against pre-existing varicella zoster antigens at the beginning of immune restoration, rather than profound immunosuppression [4,27]. However, like a few other observational studies [2,16], we did not observe such a phenomenon in this study. Possible reasons for this discrepancy might include: (1). HAART users in our study had higher CD4+ T cell counts (372 cells/μl) before starting HAART; (2). In this short term analysis, our study might not have sufficient power to detect minor difference in HZ incidence due to relatively small sample size and rarity of HZ events; (3). Participants were followed every 6 months and the exact dates of HAART initiation could not be determined.

Since HAART became available in 1996, a steady decrease in HZ incidence over time has been observed among HIV-infected women in the WIHS coincident with their increasing access to HAART. Like many other studies [18-21,28], ours showed a protective effect of long-term HAART use on HZ incidence. However, a statistically significant effect was achieved only when a HAART adherence level of ≥95% was attained. Thus, a further examination of the role of HAART adherence level might shed light on the lack of associations between HAART use and HZ incidence as reported by some earlier studies as no such information was reported [16]. In addition, we found that the long-term effect of HAART on HZ incidence was mainly mediated through its effects in reducing HIV viral load and improving host immune status. Enhanced immune status and decreased shedding of varicella zoster virus antigen by HAART [29] might explain the protective effect of HAART on HZ incidence as observed in our study.

In this study, we found that acyclovir use was surprisingly associated with higher HZ incidence in univariate analysis. Possible explanation for this lies in that prior acyclovir use might be an indication of other herpes infections that were closely related to HZ incidence due to common cause - immunosuppression by HIV infection, rather than be used to prevent occurrence of HZ [30,31]. In addition, lower overall QOL was also associated with higher incidence of herpes zoster, which is consistent with the role of psychological stress in the development of HZ [32].

Our study had some advantages over previous studies. First, we used a propensity score matching method to balance background covariates. In this way, the indication bias for HAART initiation was dramatically decreased. Second, previous studies focused on HAART use but did not take into account HAART adherence level. Our study showed the protective effect of HAART use was most clear when a ≥95% HAART adherence level was attained, suggesting that HAART adherence level should always be considered in future similar studies. However, some limitations of our study should also be mentioned. First, self-reported HZ was not documented by a health professional; however, a previous study has ascribed high validity to self-reports of HZ [33], suggesting that HZ misclassification in our study was likely to be very low. Second, we used self-reported HAART adherence as an exposure variable. However, this concern might be mitigated by the fact that the relationships between the WIHS participants and their interviewers have been built on trust and respect over many years, and previous WIHS research has shown that self-reported medication use is consistent with objective measures of HIV outcomes, such as CD4+ T-cell count, HIV viral load, and self-reported physical functioning [34]. Lastly, our study only included women, and the results might not be generalizable to men.

## Conclusion

We have shown that individuals highly adherent to HAART regimes had a significantly lower incidence of HZ than similar individuals who were not as adherent to HAART, underscoring the importance of high HAART adherence level in the clinical management of HIV-infected individuals, in addition to its role in decreasing HIV disease progression and HIV transmission.

## Abbreviations

HAART: Highly active antiretroviral therapy; HZ: Herpes zoster; HR: Hazard ratio; CI: Confidence interval; IQR: Inter-quartile range; HIV: Human immunodeficiency virus; AIDS: Acquired immune deficiency syndrome; WIHS: Women’s Interagency HIV Study.

## Competing interests

Dr. Glesby had consultancy relationship with Gilead Sciences and Pfizer. All other authors declare that they have no competing interests.

## Authors’ contributions

(Please use initials to refer to each author’s contribution): CL was responsible for study design, data interpretation and manuscript writing. CW participated in study design and statistical analysis. MJG, GD, AF, HM, TM, and RK participated in drafting and improving manuscript. MY participated in study design, coordination and drafting the manuscript. All authors read and approved the final manuscript.
